# Targeting PCSK9: a promising adjuvant strategy in cancer immunotherapy

**DOI:** 10.1038/s41392-021-00530-6

**Published:** 2021-03-06

**Authors:** Catarina R. Almeida, Beatriz H. Ferreira, Iola F. Duarte

**Affiliations:** 1 iBiMED – Institute of Biomedicine, Department of Medical Sciences, University of Aveiro, Aveiro, Portugal; 2 CICECO – Aveiro Institute of Materials, Department of Chemistry, University of Aveiro, Aveiro, Portugal

**Keywords:** Immunotherapy, Cell biology

Treatment with immune checkpoint inhibitors (ICIs) has drastically improved the prognosis of certain advanced-stage cancers. However, low response rates and the development of resistance remain important limitations. In a study recently published in *Nature*, Li et al. propose that antibody inhibitors of proprotein convertase subtilisin/kexin type 9 (PCSK9), FDA-approved for hyperlipidemia treatment, have the potential to boost the anticancer efficacy of ICIs.^1^

In a series of experiments using immunocompetent syngeneic mice and four cancer cell lines (B16F10—melanoma, 4T1—mammary carcinoma, MC38—colon adenocarcinoma, and CT26—undifferentiated colon carcinoma), the authors started by showing that tumors resulting from inoculation of PCSK9 knock out cells, obtained through CRISPR-cas9 gene editing, displayed significant growth retardation compared to wild-type counterparts. As PCSK9 regulates cholesterol homeostasis, by binding to the low-density lipid receptor (LDLR) and triggering its intracellular degradation,^2^ the role of cholesterol metabolism in this response was investigated. LDLR-deficient melanoma cells kept their ability to form tumors in syngeneic mice, and tumor growth suppression by PCSK9 deletion was still observed in LDLR-deficient mice fed a high fat diet. Additionally, lowering LDLR levels had no impact on the surface expression of major histocompatibility protein class I (MHC I) molecules, found to be critically involved in the anti-tumor effect of PCSK9 deficiency, as discussed further ahead. Therefore, the impact of PCSK9 deficiency on tumor growth was independent of cholesterol metabolism.

Inoculation of PCSK9 knock out cells into immunodeficient mice did not result in tumor growth attenuation, which hinted to the possible involvement of immune cells in the response to PCSK9 deletion. This hypothesis was corroborated by data showing that long-term survivors of the first inoculation with PCSK9-deficient tumor cells developed anti-tumor immune memory, as shown by rejection of a second inoculation with wild-type tumor cells. A putative immunomodulatory role for PCSK9, unrelated to LDL-lowering, has also been recently highlighted in the context of atherosclerosis. Liu and Frostegård have shown that silencing of PCSK9 could reverse oxLDL-induced maturation of dendritic cells (DCs) and subsequent T cell activation, while inducing regulatory T cells with IL-10 production.^3^ However, the molecular mechanisms underlying the direct influence of PCSK9 in atherosclerosis pathogenesis and regression remain to be elucidated.

Remarkably, tumor growth retardation and overall survival were further promoted upon treatment of syngeneic mice bearing PCSK9 knock out tumors with a mouse anti-PD1 antibody. This immune checkpoint inhibitor acts by blocking the interaction of the PD1 receptor on T cells with the PD-L1 ligand on tumor cells, thereby boosting T cell activation and tumor cell destruction. Authors then tested the efficacy of PCSK9 pharmacological inhibitors in enhancing the response to the anti-PD1 treatment. Evolocumab and alirocumab are anti-PCSK9 blocking antibodies in clinical use for the treatment of hypercholesteremia and cardiovascular risk reduction, due to their impact on LDLR levels. Treatment with these antibodies significantly retarded the growth of MC38 tumors and, especially when used in combination with the anti-PD1 antibody, had a positive impact on long-term survival. Importantly, anti-PD1-resistant tumors (MC38R) also responded to evolocumab-induced PCSK9 inhibition.

Lymphocytic infiltration is one of the key factors dictating tumor responsiveness to immunotherapy.^4^ PCSK9-depleted tumors displayed significantly increased numbers of tumor-infiltrating lymphocytes, with intratumoral IFN-γ^+^ CTLs showing a higher raise in response to evolocumab than upon anti-PD1 antibody treatment. Moreover, PCSK9 deficiency failed to retard tumor growth in mice depleted of CD8^+^ T cells, while depletion of CD4^+^ T cells had hardly any impact, which led CD8^+^ cytotoxic T cells (CTLs) to be identified as the most critical for the anti-tumor effect of PCSK9 deficiency. PCSK9-deficient melanoma cells were also shown to be highly susceptible to CTL killing in vitro. This was correlated with an increased expression of MHC I molecules, but not MHC II, on the surface of PCSK9-deficient tumors or upon PCSK9 antibody blockage. This effect could be compensated when PCSK9 levels were rescued with exogenous protein, showing that PCSK9 controls the surface levels of MHC I, which directly impacts the antigen presentation capacity of tumor cells. The increase in MHC I expression levels on target cells was further accompanied by an increase in the diversity of TCRs, thus translating into a higher probability of CTLs recognizing and lysing the tumor cells.

In healthy nucleated cells, MHC I proteins display endogenous peptides at the cell surface for recognition by CD8^+^ T cells. MHC I surface levels are tightly regulated to guarantee presentation of antigens for possible activation of CD8^+^ T cells, while avoiding NK cell activation. Intracellular antigens are processed for presentation in MHC I through the endogenous pathway, before the MHC I-peptide complex is transported by the secretory pathway to the cell surface. In specialized DC subsets MHC I may also present exogenous antigens that have been processed by cross-presentation pathways. MHC I-peptide complexes will display variable lifetime at the cell surface, with MHC I being internalized and recycled back to the cell membrane. The recycling pathway starts by the formation of endocytic vesicles, which are internalized into the early sorting endosomes. Internalized cargo can then be redirected to the cell surface or sorted to late endosomes or lysosomes for degradation. Li et al. found that PCSK9 interacts with MHC I and promotes its trafficking to the lysosomes and subsequent degradation, while blocking its recycling back to the membrane.^1^ As a consequence, cells expressing PCSK9 have reduced levels of surface MHC I and thus escape CTL recognition, while PCSK9 deficiency or inhibition promote tumor-T cell interaction (Fig. 1).

**Fig. 1:**
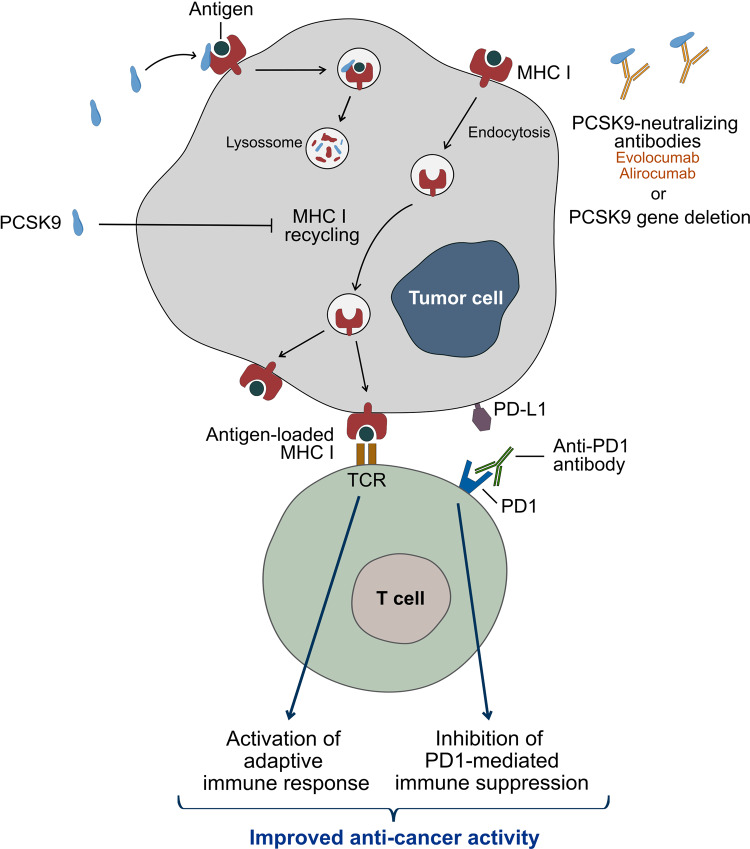
Blockage of PCSK9 with the antibodies Evolocumab and Alirocumab or PCSK9 gene deletion enable the recycling of MHC I to the tumor cell surface improving the efficacy of immune checkpoint inhibitors

Interestingly, PCSK9 trafficking to the lysosomes is also mediated by a direct interaction with amyloid precursor-like protein 2 (APLP2). Furthermore, APLP2 routes H2-K(d), an MHC I molecule, to the lysosomes upon binding to it at the plasma membrane and promoting endocytosis.^5^ These data suggest that there is a transport complex that might include PCSK9, APLP2, and MHC I, targeting surface proteins for lysosomal degradation. It has also been previously demonstrated that PCSK9 promotes degradation of other membrane receptors, including LDLR, CD81, and CD36.^2^ PCSK9 may thus regulate endocytosis of membrane microdomains, such as lipid rafts, which will be enriched in the identified receptors, and may be diverted to the lysosomal degradation pathway. Overall, this paper by Li et al. contributes to elucidate the mechanisms regulating MHC I recycling, while also exploring the possibility of targeting MHC levels in combination with ICIs as a means to develop more effective anticancer immunomodulatory strategies.

## References

[1] Liu X (2020). Inhibition of PCSK9 potentiates immune checkpoint therapy for cancer. Nature.

[2] Liu X (2019). The immune functions of PCSK9: Local and systemic perspectives. J. Cell Physiol..

[3] Liu A, Frostegård J (2018). PCSK9 plays a novel immunological role in oxidized LDL-induced dendritic cell maturation and activation of T cells from human blood and atherosclerotic plaque. J. Intern Med.

[4] Paijens, S. T., Vledder, A., de Bruyn, M. & Nijman, H. W. Tumor-infiltrating lymphocytes in the immunotherapy era. *Cell. Mol. Immunol*. 10.1038/s41423-020-00565-9 (2020). 10.1038/s41423-020-00565-9 PMC8115290 33139907

[5] DeVay RM, Shelton DL, Liang H (2013). Characterization of proprotein convertase subtilisin/kexin type 9 (PCSK9) trafficking reveals a novel lysosomal targeting mechanism via amyloid precursor-like protein 2 (APLP2). J. Biol. Chem..

